# Retraction: Loss of CCDC6, the First Identified RET Partner Gene, Affects pH2AX S139 Levels and Accelerates Mitotic Entry upon DNA Damage

**DOI:** 10.1371/journal.pone.0359046

**Published:** 2026-09-24

**Authors:** The PLOS One Editors

Following the publication of this article [1] concerns were raised regarding results presented in Figs 2-3, and Figs 5-7. Specifically,

- There appear to be irregularities in the following figures;
  - Fig 2e, Sh CTRL IB anti-myc NT γH2AX.
  - Fig 5b CCDC6 (left panel).
  - Fig 5f WCL siRNA Tubulin.
  - Fig 5d PP4c.
  - Fig 6d IP anti-PP4c panel.
- The following bands appear more similar than would be expected from independent results:
  - Fig 2f pS1981-ATM lanes 5 and 8.
  - Fig 3d Sh CTRL pS317Chk1 lanes 2-3 and Fig 3d Sh CTRL Chk1 lanes 3-4.
  - Fig 5b PP4c (right panel) lane 2 and Fig 5f IP anti-CCDC6 siRNA PP4c lane 2.
  - Fig 5b PP4c (right panel) lane 3 and Fig 5f WCL siRNA PP4c lane 1 when flipped.
- There appear to be irregularities in the background noise of the Fig 2f panels γH2AX and pS1981-ATM.
- The following panels appear similar, despite being used to represent different results:
  - Fig 2e Sh CCDC6 IB anti-myc ETO 2.5 μM H2AX and Fig 5f WCL siRNA PP4c.
  - Fig 5a WCL Myc (bottom panel – lanes 3-4) of [1] and Fig 6B WCL myc CCDC6 (bottom panel) of [2].
  - Fig 5b CCDC6 (right panel) of [1] and Fig 6A CCDC6 of [2].
  - Fig 6d H2AX and Fig 6d WCL Tubulin when flipped.
  - Fig 7b Sh CRTL RPA2 and Fig 7b Sh CCDC6 RPA2 when flipped.

The corresponding author provided the original underlying blot for the Fig 2e Sh CTRL IB anti-myc NT γH2AX panel which confirmed that this panel was prepared from a spliced blot. In addition, the corresponding author provided the underlying data for the Fig 5f PP4c panels and the Fig 6d H2AX panel, which support these results.

Regarding the panel duplication between Fig 5a of [1] and Fig 6B of [2], the corresponding author stated that these panels are intentionally duplicated as they represent the same experiments. However, they clarified that there is an error in the labelling of the Fig 6B panel of [2]. The underlying data for these blots were not provided for editorial review.

Regarding the panel duplication between Fig 5b of [1] and Fig 6A of [2], the corresponding author stated that these panels are intentionally duplicated as they represent the same experiments. However, their explanation does not resolve the concern that the labels in both articles suggest different conditions for lanes 2 and 3, and it remains unclear which panel is correct. The underlying data for these blots were not provided for editorial review.

The original blots underlying the remaining panels of concern are no longer available. In the absence of the original image data, the concerns involving these figures cannot be fully resolved.

In light of the unresolved concerns, which call into question the reliability and integrity of the published results, the *PLOS One* editors retract this article.

FM and AC did not agree with the retraction. CL, MTM, RP, and AF either did not respond directly or could not be reached.
